# A Digital Nudge to Counter Confirmation Bias

**DOI:** 10.3389/fdata.2019.00011

**Published:** 2019-06-06

**Authors:** Calum Thornhill, Quentin Meeus, Jeroen Peperkamp, Bettina Berendt

**Affiliations:** Department of Computer Science, KU Leuven, Leuven, Belgium

**Keywords:** digital nudging, fake news, confirmation bias, NLP (natural language processing), Twitter

## Abstract

Fake news is increasingly an issue on social media platforms. In this work, rather than detect misinformation, we propose the use of nudges to help steer internet users into fact checking the news they read online. We discuss two types of nudging strategies, by presentation and by information. We present the tool BalancedView, a proof-of-concept that shows news stories relevant to a tweet. The method presents the user with a selection of articles from a range of reputable news sources providing alternative opinions from the whole political spectrum, with these alternative articles identified as matching the original one by a combination of natural language processing and search. The results of an initial user study of BalancedView suggest that nudging by information may change the behavior of users towards that of informed news readers.

## 1. Introduction

Information disorder in current information ecosystems arises not only from the publication of “fake news,” but also from individuals' subjective reading of news and from their propagating news to others.

Sometimes the difference between real and fake information is apparent. However, often a message is written to evoke certain emotions and opinions by taking partially true base stories and injecting false statements such that the information looks realistic. In addition, the perception of the trustworthiness of news is often influenced by confirmation bias. As a result, people often believe distorted or outright incorrect news and spread such misinformation further.

For example, it was shown that in the months preceding the 2016 American presidential election, organizations from both Russia and Iran ran organized efforts to create such stories and spread them on Twitter and Facebook (Cohen, 2018).

It is therefore important to raise internet users' awareness of such practices. Key to this is providing users with means to understand whether information should be trusted or not.

A solution put forward by social networks relies on users identifying suspicious articles shared on their platforms. Such articles are subsequently fact-checked by third-party volunteers. Then, when another user comes across such an article, they are given the chance to read an alternative article that has been deemed trustworthy.

However, this method is labor-intensive and requires highly skilled humans and therefore does not scale. In addition, important fact-checking organizations have become disillusioned by social networks' handling of the “fake news” problem and of their fact-checking efforts, and have withdrawn their support (Lee, 2019).

In this work, we propose BalancedView, a novel, low-cost, and scalable method for fighting the spread of misinformation without having to rely on users reporting or third parties checking news items.

The method presents the user with a selection of articles from a range of reputable news sources providing alternative opinions from the whole political spectrum, with these alternative articles identified as matching the original one by a combination of natural language processing and search. The strategy is a form of digital nudge, in which the user is presented with an original text together with articles showing wider context and alternative standpoints within close view.

Our main objective for such a tool is to educate people about sharing and believing information accessed online, which in turn can decrease the spread of fake news. We also hope to raise awareness of the different ways information can be presented and manipulated online.

In section 2, we briefly discuss the mechanisms of misinformation spreading online and how social networks are the perfect platforms to accelerate this process, and we give a brief overview of related research in the field of fake news and nudge design. section 3 gives a high level description of our approach. In section 4, we discuss the nudging strategies considered.

Technical design and the inner workings are covered in section 5, along with first evaluations of algorithm and user assessments in section 6. We conclude with an outlook on future research.

## 2. Background and Related Work

### 2.1. Online Spreading of Misinformation

In this section, we discuss how social networks increase the spread of biased news and misinformation. We discuss confirmation bias, echo chambers and other factors that may subconsciously influence a person's opinion. We show how these processes can interact to form a vicious circle that favors the rise of untrustworthy sources.

Often, when an individual thinks they know something, they are satisfied by an explanation that confirms their belief, without necessarily considering all possible other explanations, and regardless of the veracity of this information. This is **confirmation bias** in action. Nickerson (1998) defined it as the tendency of people to both seek and interpret evidence that supports an already-held belief.

An **echo chamber** is a situation in which an individual can only hear echoes of things that have already been said (Garimella et al., 2018). Social networks such as Twitter and Facebook are environments that favor the creation of such chambers (Knobloch-Westerwick and Kleinman, 2012). People tend to mix with others who think like them and follow news sources that they favor. In so doing, they expose themselves to limited framing of events that obscures other perspectives for them.

Consider a user with a hard-line political belief on either side of the political spectrum. They may follow only people and news sources who share that belief. It is likely that upon publishing a tweet about a new policy or event, they would see similar tweets from their friends and receive feedback that favors their own opinion. The echo chamber around the user shelters them against conflicting opinions. The 2016 American presidential election illustrates this phenomenon very well. Donald Trump's victory came as a surprise to many people worldwide. One explanation of this surprise is that voters on either side of the political spectrum were enclosed in echo chambers.

Research and having a critical approach to information shared online can protect a user against biased views, but very few protections exist against the creation of echo chambers. People can learn to identify them, but to avoid them completely requires them to ensure that all opinions are represented within their social circle.

Social networks extensively use recommender systems algorithms for selecting the content that appears in the feeds of users (Chakraborty et al., 2016). The reason is simple: the amount of content being created is too large for any single person to keep track of. Also, social networks want to improve the user experience by displaying content that the user will appreciate. This only exacerbates the problems discussed as it implies that users are grouped into clusters of preferences and provided with filtered content.

These recommender systems rely mostly on artificial intelligence to decide which content is best for a particular user (Ricci et al., 2011). Whether they are based on content-based filtering, on collaborative filtering, or on hybrid models, they tend to provide users with more content similar to that already seen and deemed relevant by and for similar people—thus enabling confirmation bias and feeding echo chambers.

Indeed, this is a key part of the functionality of the platforms: users are provided with content that they will like by restricting material that may not encourage further interaction.

These phenomena together can create a vicious circle. Echo chambers arise from both the user's subconscious choice of surrounding themselves with like-minded people and the enticement by content presented by recommender systems. Viewing a limited framing of content further increases the confirmation bias that what they believe is right. Finally, when users respond to articles that they “like,” they close the loop by feeding the recommender algorithms that provide them with content.

### 2.2. Approaches to Detecting and Fighting Fake News

Lazer et al. (2018) argue that a scientific approach is required to find a solution to fake news in social media. Homogeneous social networks allow polarization and closure to new information. Consequently, echo chambers can form because of the personalization of political information. An additional reason for their formation is linked to both human behavior and the technical foundations of the user experience.

Despite the intellectual high ground taken by fact checkers such as PolitiFact^1^ and Snopes^2^, they do not solve the issue that is the tendency of individuals not to question the veracity of sources unless their own values or beliefs are infringed. This suggests that it is unlikely that a user would actively engage in the fact checking process and use the services provided by these fact checkers. Instead, the authors argue that it is the responsibility of platforms to include signals as to the quality of a source or article within their algorithm, for example the prioritization of reputable sources in the news feed. However, this does not solve misinformation or ensure that conflicting views are available to the user. Methods for addressing these problems are still lacking.

### 2.3. Digital Nudges

The day-to-day definition of nudge as defined by Thaler and Sunstein (2008) is “*to push mildly or poke gently in the ribs, especially with the elbow*” or, applied to an economical context, “*self-consciously [attempt to] move people in directions that will make their lives better*.” In a digital world, the definition is no different: the idea is to influence someone's behavior into acting in such a way that will improve his or her user experience and/or choices.

Lazer et al. (2018) cite nudges as a reasonable solution to the problem laid out in section 2.2. If the reading of news on social media platforms without investigating alternatives is re-framed as a choice for belief without validation, it is possible to define an architecture around this choice. Thus, it is possible to adapt this architecture through implementation of a nudge.

Several researchers have put effort into understanding the impact of nudges in social media, including Acquisti et al. (2017) and Wang et al. (2014), who have considered nudges to encourage user awareness of privacy and the impact of posts on platforms. Acquisti et al. (2017) discuss nudging by means of information, presentation, defaults, incentives, reversibility, and timing. We summarize two of these strategies for nudges here: nudging with information and presentation.

Nudging with information involves providing information to raise awareness. For example, in the context of fake news, this may include giving a label or signal about the reputability or the political leaning of a source.

Nudging with presentation involves the framing and structure of a choice. In the context of reading news in social media, an example could be the placement of an article in relation to the story from across the political spectrum.

## 3. BalancedView: An Approach to Mitigate Online Bias and Misinformation

In the context of fighting confirmation bias and fake news in the Twitter news feed, several approaches can be imagined, e.g., removing all suspicious posts. Another example would be to not allow users to post political views that are judged to be too extreme. This second example reduces the platform's usability. Instead, a solution must be more subtle and not restrict a user from posting or reading any particular post. In the present section, we give an overview of our approach.

We propose the approach and tool *BalancedView*^3^ that aims to encourage users to consider the wider view surrounding information. In a first proposal, we will focus on tweets from the well-known social platform Twitter. We aim to implement a tool that efficiently presents a full view on articles from relevant sources presenting opinions from everywhere in the political spectrum. Practically, a user would input a tweet and be shown articles from trustworthy sources reporting on the same topic but with different opinions.

By doing so, a user is given the opportunity to forge their own opinion by reading from multiple sources. They can then make an informed decision on whether to believe an article based on presented alternatives. The proposed nudge is equivalent to placing the healthier bananas at eye level alongside an unhealthier option. The aim of the nudge is to ensure that a reader of a post is not restricted to reading the original content and is instead given a balanced view of the information based on sound journalism. Rather than restrict content and usability, we place a balanced and reputable selection of news sources at eye level to a news item.

A user can input a tweet to the tool, which then extracts the relevant text to structure a query to an API of news sources. Afterwards, the user is presented with the alternative framing of the same information. In further work, this system will be embedded into the user experience within Twitter.

This choice architecture corresponds to the “nudging by presentation” strategy of Acquisti et al. (2017) (see the overview in section 2.3). We also compared this with the strategy of nudging by information, which is closer to the approach currently taken by Twitter itself. The design of these two nudges will be described in the next section. Section 5 will then detail the back-end processing that identifies the appropriate news articles with alternative framing.

## 4. Two Nudging Strategies

The desired outcome for a user should be an increased awareness of the potential political bias in an article. Subsequently, this should bring about assessment of evidence and consideration for how bias may compromise the veracity of an article.

We focus on tweets posted on Twitter and discuss two approaches, nudging by presentation and nudging by information as proposed by Acquisti et al. (2017). In the former case, the user is directly presented with information that might affect their judgement. In the latter case, a visual cue is displayed that gives the user an idea about the veracity of information. Both approaches follow the development process for digital nudges proposed by Mirsch et al. (2017):

- **Define**: The context is defined as the news feed of a social media platform. In the environment, only one-sided opinions are visible in the personalized sources chosen by a user. The goal is to ensure that at all times, without restricting a user from viewing the original content, the user is encouraged to view a balanced representation of opinions on a subject.
- **Diagnose**: In understanding the decision process, a number of questions can be identified that would ideally be asked by any reader of news such that a reasonable investigation of reputability and veracity of source or story is made. That is, given the set of questions that a professional fact checker would ask, is there a change in the choice architecture that would encourage a non-fact checker to ask similar questions.
- **Select**: For the scope of this work, we selected a nudge by presentation and a nudge by information. These strategies are explained in detail below.
- **Implement**: The nudges should be embedded in the social media platform on which people read news, but the exact HCI choices should be designed on the basis of a formative evaluation. We therefore implemented a mock-up Twitter interface for the user test. In addition, an emulated environment with a web front end was created for testing the natural language processing required for the nudge. More information on back-end processing for the two nudging strategies is given in the following two subsections.
- **Measure**: The nudge was evaluated by means of an initial user study. In a survey, users were asked to rate an article based on perceived levels of truth and reputability, in the presence and absence of nudges. This is discussed in section 6.

### 4.1. Nudging by Presentation

The primary aim of the nudge is to present an unbiased view of a subject, without necessarily forcing a user to embrace it. The secondary aim, of equal importance to the first, is to ensure that the sources presented are of a sufficient level of reputability: even if occasionally headlines are sensationalized, the underlying article will not be entirely fictitious or propagandistic.

#### 4.1.1. Select the Appropriate Nudge

The objective is to present a user with alternative information that should encourage judgement of the veracity of a news article.

#### 4.1.2. Implementation

Natural language processing is used to extract meaning from a tweet, and an API of news sources, NewsAPI ^4^, is queried. These results are sorted by relevance and presented to the user. This is described from a high-level perspective in section 3 and discussed in more detail in section 5.

#### 4.1.3. Presenting the Nudge

**Figure 1:**
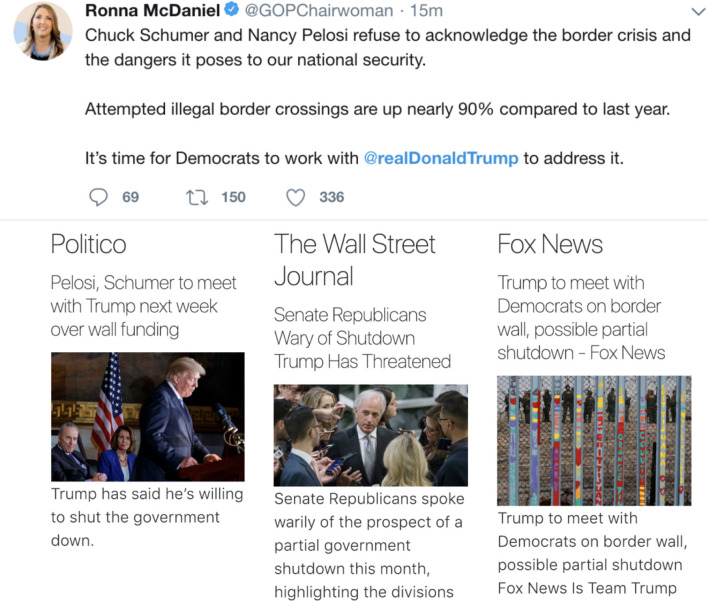
Placement of the nudge relative to original text.

The alternative news sources are displayed directly below the original content. This does not restrict the user from reading the original content but achieves the purpose of placing the alternative view at eye level. This is shown in Figure 1.

#### 4.1.4. Selecting News Sources

A key aspect to discuss is how the sources are selected. The perceived political affiliation of news sources is identified through reports from Pew Research (Mitchell et al., 2014) and YouGov (Smith, 2017). Based on their findings, we chose the news sources shown in Table 1 for use in the tool.

**Table 1:** A selection of trustworthy news providers.

**Left**	**Center**	**Right**
	Reuters	
The Guardian	The Financial Times	The Telegraph
Independent	BBC News	The Daily Mail
MSNBC	The Wall Street Journal	Fox News
Politico	CNN Bloomberg	

### 4.2. Nudging by Information

The second approach aims to provide information to raise awareness. For example, in the context of fake news, this may include giving a label or signal about the reputability of a source or its political bias. Twitter has implemented this to some extent by classifying some accounts as “verified.” The existing Twitter flag for verified accounts can be regarded as a nudge towards trusting a source. Building on this format familiar to Twitter users, we have designed a nudge to encourage users to question a source. This nudge consists of a small white cross surrounded by a red background. It does not necessarily suggest bias or lack of reputability but it is the antithesis of the current nudge. In the study reported in section 6, we tested only the more well-known Twitter flag for verified accounts.

## 5. Analyzing an Article and Identifying Alternatives with Different Framing

In this section, we describe the back-end behind BalancedView's nudging by presentation.

### 5.1. High-Level Description

When a user inputs a tweet, the system first extracts and summarizes into relevant keywords the information contained in the text using the TextRank algorithm (Mihalcea and Tarau, 2004). With the keywords, the system builds a query to search for articles using the NewsAPI. Articles from multiple sources are then displayed on the screen ranging from left-most to right-most view. The selection of news providers is discussed in section 4.

### 5.2. Overview

The system takes a text as input and displays a series of articles, sorted by relevance and by political affiliation. We have separated this process into three main steps: summarizing the input, querying the news providers, and displaying the results by categories.

#### 5.2.1. Summarizing the Input

In order to be able to query news providers, it is necessary to summarize the input and extract only the keywords. Among the relevant algorithms, TextRank and its variants provide a simple method based on a strong theoretical ground (Mihalcea and Tarau, 2004; Barrios et al., 2016). This algorithm performs unsupervised identification of centrality of text, using pre-trained models for the low-level tasks like part-of-speech tagging and stemming, as well as graph-based models for the identification of relevant entities.

When the algorithm receives an input, it tokenises the text and removes stop words, numbers and punctuation as well as Twitter-specific keywords such as hash tags and user mentions. The remaining words go through a part-of-speech filter and only the nouns, adjectives and verbs are kept. Porter's stemmer (Porter, 1980) is then used to generalize the words further.

From there, the algorithm builds a graph where each token is a node and the edges represent the relations between them. An edge between two words denotes that these two words follow each other in the text. A scoring function assigns scores to each node based on the nodes that are reachable from the first word of the input text. In other words, any words for which a path can be found from the starting node will have a high score. Consequently, words that occur repeatedly or that occur after such repeated words are more likely to have a high score and words that occur only once at the end of the input will have a low score.

**Figure 2:**
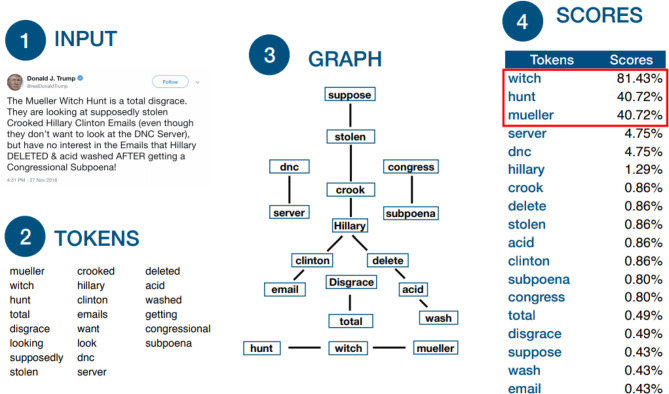
Graph construction: (1) The input text from Twitter. (2) Token extraction based on the Porter stemmer (Porter, 1980). (3) A graph is constructed where nodes are words and edges denote whether two words are juxtaposed in the text. (4) Scores reflect whether a node is reachable from the start of the input text. If more than 5 words reach a score of 10%, only the best 5 keywords are selected. If less than 3 keywords reach this threshold, the 3 best keywords are selected.

Next, the keywords are sorted by decreasing score and the three to five best keywords are kept for the next step. The selection is based on a minimum score of 10%. Both the optimal number of keywords and the minimum score were empirically selected based on the quality and quantity of results after querying the source providers. The whole process described above is depicted in Figure 2.

For the proof-of-concept, we deliberately chose this simple method for the initial testing of the approach set forward in this work. In the future it should be improved to increase robustness to the shorter text lengths used on Twitter and other platforms.

#### 5.2.2. Querying the News Providers

Having identified the keywords, a query is built and sent to NewsAPI. This service allows us to query a plethora of sources at the same time and get results from a number of countries in multiple languages. However, the free version does not enable going back more than one month in the past, which limits the number of results. The sources selection is explained in section 4 and the sources are listed in Table 1.

#### 5.2.3. Displaying the Results

As we have discussed in the previous sections, the nudge must be subtle and cannot overload the user with too much information. Consequently, the design must be clean: the two most relevant articles for each political affiliation are included and only an abstract of the articles is displayed, together with a photo when one is available.

## 6. Evaluation

### 6.1. Relevance of the News Articles Presented

From a set of 35 tweets covering a number of stories in American and British politics, a query was built and evaluated. Such a test was deemed successful if at least two of the articles presented first in the results were considered relevant to the news surrounding the query.

Relevance was rated by the first two authors of the current paper. Their relevance ratings coincided in all 35 cases. Out of the 35 trials, all three articles were relevant eighteen times, two out of three were relevant eleven times, and in six cases, the system returned one or no relevant articles.

### 6.2. Effectiveness of the Nudge: User Study

We tested the usefulness of nudging by presentation and information in the context of perception of news. These experiments were made in survey form, in which participants were presented with tweets and asked to rate them on both impartiality and trustworthiness.

#### 6.2.1. Method

We recruited twenty participants via an advertisement on our university's degree programme's Facebook page that contained a link to a survey. All participants were Master students of Artificial Intelligence, and they are regular users of social media including Twitter. No further demographic information was collected. Participation was voluntary and unpaid. Only aggregate results were retained.

We created a survey to test whether the nudge was effective in lowering trust in an intentionally selected politically biased tweet. Furthermore, the survey questioned whether the feature of a visual cue is useful in encouraging users to question reputability of a source.

The survey consisted of five individual web pages, each of which contained a screenshot of a tweet, enhanced (for questions 2, 3, and 5) by one of the two types of nudges tested, and a question regarding the trust in the news source or information.

For better readability, these questions are listed in third-person form as our research questions here; participants received a second-person “you” question. An example is shown in Figure 1. A PDF version of all survey questions is available as an online supplement to the current paper ^5^.

1. Do people trust obviously disreputable news sources in the absence of a nudge by information? Here a participant is presented with news from a disreputable news source on a news story that does not provoke an emotional response.
2. Do people trust obviously reputable news sources in the presence of a nudge by information? A participant is presented with a story from a highly reputable news source, such as the BBC.
3. Do people trust news sources of questionable reputability in the presence of a nudge by information? A participant is presented with a story from a verified news source that is unlikely to be known as reputable or disreputable.
4. Do people consider politically biased information a fair representation of a view, in the total absence of nudges?
5. Do people consider politically biased information a fair representation of a view, given a nudge by presentation? The participant is presented with a politically biased statement and linked article, in the presence of the nudge designed in this work.

#### 6.2.2. Results and Discussion

**Figure 3:**
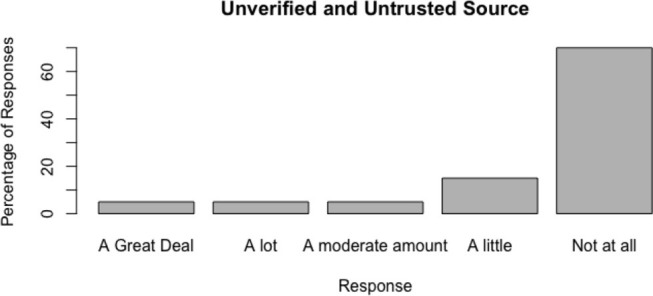
Do people trust obviously untrustworthy news sources in the absence of a nudge by information?.

**Figure 4:**
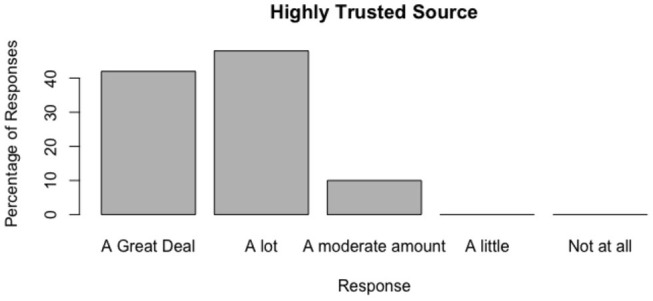
Do people trust obviously reputable news sources?.

**Figure 5:**
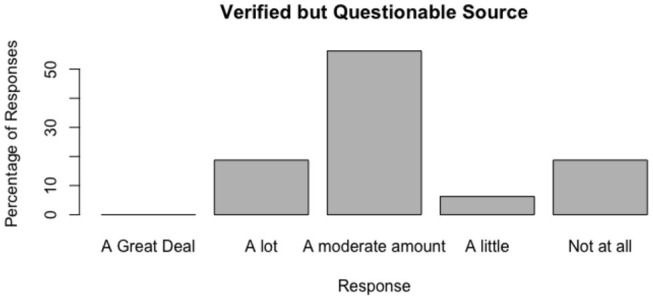
Do people trust news sources of questionable reputability in the presence of a nudge by information?

**Figure 6:**
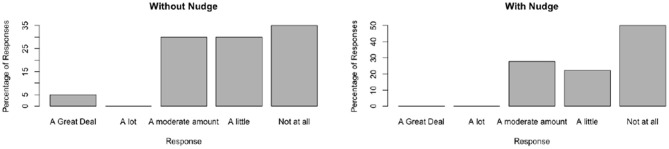
Nudge comparison.

The distributions of responses are shown in Figures 3–6.

#### 6.2.3. Do People Trust Obviously Untrustworthy News Sources in the Absence of a Nudge by Information?

Trust for the news source was generally low. Responses were concentrated in showing distrust or severe distrust of the news source. However, 15 percent of respondents placed moderate to high trust in the source despite no verification of the account.

#### 6.2.4. Do People Trust Obviously Reputable News Sources?

There was a positive result for this test, people generally tended to trust or highly trust these sources. All respondents trusted the source moderately to highly.

#### 6.2.5. Do People Trust News Sources of Questionable Reputability in the Presence of a Nudge by Information?

The results for this test were evenly spread between trusting and not trusting the source. The account was verified and the article featured was produced by a reputable news outlet. The spread of responses shows more trust than in the case of the obviously disreputable source, however, less trust is evident than in the case of the highly reputable source.

#### 6.2.6. Do People Consider Politically Biased Information a Fair Representation of a View, Given a Nudge by Presentation? Do People Consider Politically Biased Information a Fair Representation of a View, in the Absence of a Nudge by Presentation?

The key test of the effectiveness of the balanced view nudge is the change in results for questions four and five. From the limited sample size, there is a visible shift in the responses to placing less trust in the singular view.

Initially, positive trust was placed in the fairness of the view being given. In the presence of the nudge, this opinion changed. In this case, results showed that people generally thought the view was unbalanced.

In sum, the results of this initial user study suggest that users generally recognisee obviously untrustworthy news sources, and that nudging by information may influence trust judgements less than a source's obvious reputability. There is evidence that the nudge by presentation, i.e., the central idea of BalancedView in which the user is offered a spectrum of diverse articles, helps participants question the trustworthiness of politically biased information.

Nonetheless, the survey questions need further development. The first questions in the current study were intended as a “sanity check” of intuitions about user trust in reputable and non-reputable sources, and about the basic workings of a nudge. The results support these intuitions and allow us to proceed to the more involved later questions. The results of the latter also show our user sample to be quite critical from the start, which may result in a ceiling effect in that nudges do not significantly change users' perceptions. In future work, more diverse groups of users should be drawn upon, such that the nudges' possible effects on their perceptions and actions become clearer.

## 7. Summary, Limitations, and Future Work

We have discussed nudges as a solution approach to the combined effects of confirmation bias and the algorithms of social media platforms that may create echo chambers and feedback loops of misinformation. This involves gently steering users towards adopting fact checking habits in their behavior online. Two nudging strategies were proposed: one that presents results in a way that pushes the user to look further and another that gives feedback on the quality of the posts that are shared online. The former option was implemented into an online tool that can be used to quickly browse articles relating to information expressed in a short text such as a tweet. The articles come from trustworthy news providers and are classified into political categories. In summary, the tool can be used to quickly and efficiently fact check any piece of information that one might read online.

In an initial user study, we investigated how questionable articles were perceived without any nudging strategy and with one of the two approaches discussed. The results suggest that the nudging strategies make people more aware of the trustworthiness of the sources. Furthermore, there is potential in presenting a balanced view of related news as a solution to lowering acceptance of a singular view.

These findings are encouraging. However, future work is needed to address a number of limitations:

- **Choice of methods and algorithms:** *BalancedView* in its current version uses relatively simple methods; our goal was to leverage the extensive toolbox of natural language processing and search algorithms for a new and timely purpose. We built this first version of our tool in order to establish a baseline from which to explore, in the future, different methods and algorithms with regard to their specific contributions to the task of countering confirmation bias.
- **Importance of the first words:** *BalancedView* gives more importance to words for which a path can be built starting at the first word of the graph. Consequently, the structure of the input tweet affects the relevance of the results.
- **Spelling and abbreviation:** The part-of-speech tagger used to identify relevant information is not robust to spelling errors and out-of-vocabulary words. This affects the relevance of the results as well.
- **Time-limited results:** The free version of the NewsAPI only returns results that are less than one month old. Consequently, texts referring to older events might not generate any results.
- **Limited number of sources:** The number of trusted sources should be increased, for example to reflect a wider range of political views. For this, there is a need for research in the field of political source trustworthiness.
- **Evaluation:** We have presented the results of a first relevance test and of an initial user study. Both evaluations were small-scale and need improvement along a number of dimensions. In particular, future studies should rest on larger sample sizes (both of article sets and of human participants) and experimental designs that allow for more fine-grained comparisons and contrasts between the choice architectures, and which take into account further factors such as demographics, as well as order effects.

In addition, extensions of the current approach are possible, including:

- **Multilingual Support:** Although this falls out of the scope of this project, we note that being able to not only search for tweets in any languages but also comparing information from different countries would be beneficial for the tool.
- **Deep Learning:** Recent developments in Deep Learning apply to text summarization as well as other of the limitations listed above and we think that using attention mechanisms and recurrent neural networks would help generate better results.
- **Fine-grained analysis of usage:** This can include recording interactions in the user experience, for example measuring how much time the users spend on the page and whether they still share and propagate unreliable news after having been in contact with *BalancedView*.

## Data Availability

The raw data supporting the conclusions of this manuscript will be made available by the authors, without undue reservation, to any qualified researcher.

## Author Contributions

CT: idea originator, first author and developer. QM: developer, second first author. CT and QM developed this as a project under the guidance of JP and BB, who worked as third and fourth authors to help refine the idea and write the paper in the later part of the project.

### Conflict of Interest Statement

The authors declare that the research was conducted in the absence of any commercial or financial relationships that could be construed as a potential conflict of interest.

## Footnotes

^1^https://www.politifact.com/

^2^https://www.snopes.com/

^3^https://fact-checker.herokuapp.com. The tool is currently hosted on a free server meaning a slight delay in the initial start up.

^4^https://newsapi.org

^5^https://people.cs.kuleuven.be/~bettina.berendt/BalancedView/BalancedView-Survey.pdf
